# NEDDylation antagonizes ubiquitination of proliferating cell nuclear antigen and regulates the recruitment of polymerase η in response to oxidative DNA damage

**DOI:** 10.1007/s13238-017-0455-x

**Published:** 2017-08-22

**Authors:** Junhong Guan, Shuyu Yu, Xiaofeng Zheng

**Affiliations:** 1 State Key Lab of Protein and Plant Gene Research, School of Life Sciences, Peking University, Beijing, 100871 China; 2 Department of Biochemistry and Molecular Biology, School of Life Sciences, Peking University, Beijing, 100871 China

**Keywords:** NEDDylation, ubiquitination, PCNA, oxidative stress, DNA damage response

## Abstract

NEDDylation has been shown to participate in the DNA damage pathway, but the substrates of neural precursor cell expressed developmentally downregulated 8 (NEDD8) and the roles of NEDDylation involved in the DNA damage response (DDR) are largely unknown. Translesion synthesis (TLS) is a damage-tolerance mechanism, in which RAD18/RAD6-mediated monoubiquitinated proliferating cell nuclear antigen (PCNA) promotes recruitment of polymerase η (polη) to bypass lesions. Here we identify PCNA as a substrate of NEDD8, and show that E3 ligase RAD18-catalyzed PCNA NEDDylation antagonizes its ubiquitination. In addition, NEDP1 acts as the deNEDDylase of PCNA, and NEDP1 deletion enhances PCNA NEDDylation but reduces its ubiquitination. In response to H_2_O_2_ stimulation, NEDP1 disassociates from PCNA and RAD18-dependent PCNA NEDDylation increases markedly after its ubiquitination. Impairment of NEDDylation by *Ubc12* knockout enhances PCNA ubiquitination and promotes PCNA-polη interaction, while up-regulation of NEDDylation by NEDD8 overexpression or NEDP1 deletion reduces the excessive accumulation of ubiquitinated PCNA, thus inhibits PCNA-polη interaction and blocks polη foci formation. Moreover, *Ubc12* knockout decreases cell sensitivity to H_2_O_2_-induced oxidative stress, but NEDP1 deletion aggravates this sensitivity. Collectively, our study elucidates the important role of NEDDylation in the DDR as a modulator of PCNA monoubiquitination and polη recruitment.

**Electronic supplementary material:**

The online version of this article (doi:10.1007/s13238-017-0455-x) contains supplementary material, which is available to authorized users.

## INTRODUCTION

Genome integrity is a basic requirement for cell growth, proliferation, and development. Under different exogenous or endogenous stresses, such as those caused by ionizing radiation, ultraviolet (UV) radiation, DNA-damaging chemical agents, and other oxidative chemicals, genome integrity and cell viability are greatly threatened (Jackson and Durocher, 2013). In response to these stresses, various signaling pathways involved in multiple repair mechanisms are induced, including the DNA damage response (DDR) that recruits a series of proteins to damaged DNA sites and triggers checkpoint signaling or essential repair steps (Hoeijmakers, 2001; Harrison and Haber, 2006; Branzei and Foiani, 2008; Bergink and Jentsch, 2009).

Recent studies have reported that histones H4 and H2A are substrates of NEDD8, and histone NEDDylation is involved in the DDR pathway (Ma et al., 2013; Li et al., 2014; Brown et al., 2015). NEDDylation is reported to regulate the decision between the non-homologous end joining (NHEJ) pathway and different homologous recombination (HR) subpathways by controlling CtBP interacting protein (CtIP)-mediated DNA resection (Jimeno et al., 2015). NEDD8 has the highest similarity with ubiquitin among all the ubiquitin-like proteins (Kamitani et al., 1997), and NEDDylation occurs via a similar process with different enzymatic ligases conjugating NEDD8 to the lysines of substrates (Rabut and Peter, 2008; Hochstrasser, 2009; Huang et al., 2009a; Wei et al., 2012; Zhou et al., 2016). The proteins of the Cullin family, p53, p73, BCA3, TβRII, E2F-1, ML3, AXR1 and ribosomal proteins have been identified as NEDD8 substrates (Ohh et al., 2002; Xirodimas et al., 2004; Gao et al., 2006; Watson et al., 2006; Xirodimas et al., 2008; Loftus et al., 2012; Mahata et al., 2012; Aoki et al., 2013; Hakenjos et al., 2013; Zuo et al., 2013; Zhang et al., 2014; Enchev et al., 2015; Lan et al., 2016; Mergner et al., 2017). However, compared to those of ubiquitination, the substrates of NEDD8 remain largely unknown, especially those in the DDR pathway.

Mounting evidence indicates that the ubiquitin system plays a critical role in major DDR pathways (Kirkin and Dikic, 2007; Jackson and Durocher, 2013), and one of the classic DDR pathways controlled by ubiquitination is dependent on PCNA (Hoege et al., 2002; Moldovan et al., 2007; Bergink and Jentsch, 2009). PCNA can form a homotrimeric ring-shaped structure that encircles DNA and acts as a clamp to assist DNA polymerase in mediating DNA replication (Moldovan et al., 2007). Strikingly, PCNA can be modified by ubiquitin on the conserved lysine residue (K164) and induce post-replication repair (PRR) when DNA is damaged by UV irradiation, some chemical agents, or oxidative stress (Hoege et al., 2002; Zlatanou et al., 2011). PRR is a type of DNA repair mechanism that uses translesion synthesis (TLS) or the error-free template switching pathway to resolve the DNA lesions encountered by DNA polymerase (Bergink and Jentsch, 2009; Sale et al., 2012). When PCNA is monoubiquinated by RAD6B-RAD18 E3 ligase, it can promote the recruitment of TLS polymerases like polη and mediate DNA synthesis across the bulky lesions (Kannouche et al., 2004; Bienko et al., 2005). Because many TLS polymerases have an ubiquitin-binding domain and PCNA-interacting-protein box, they can bind to monoubiquitinated PCNA specifically and ensure TLS (Kannouche et al., 2001; Kannouche et al., 2004; Bienko et al., 2005). Besides, a recent study showed that PCNA monoubiquitination has no effects on the binding affinity of polη, raising a debate on the function of PCNA monoubiquitination (Hedglin et al., 2016). Moreover, other studies have shown that yeast PCNA is modified by small ubiquitin-like modifier (SUMO) on Lys164 and Lys127, and SUMOylated PCNA can recruit a helicase Srs2 to prevent undesired recombination (Pfander et al., 2005). The crosstalk between ubiquitin and SUMO indicates that these two modifiers can compete for the substrate (Papouli et al., 2005). Recent research has shown that PCNA is also modified by ISG15, and ISGylation of PCNA recruits USP10 to deubiquitinate PCNA, thereby regulating PCNA ubiquitination under UV irradiation-induced stress (Park et al., 2014). However, whether other ubiquitin-like modifiers are involved in the switchboard among different modifications of PCNA remains incompletely understood.

In this study, we report that PCNA is a substrate of NEDD8. RAD18 functions as the E3 ligase and NEDP1 acts as its deNEDDylase. In response to hydrogen peroxide (H_2_O_2_)-induced oxidative stress, NEDDylation plays a pivotal role in the recruitment of polη by regulating PCNA monoubiquitination.

## RESULTS

### Modification of PCNA by NEDD8

**Figure 1:**
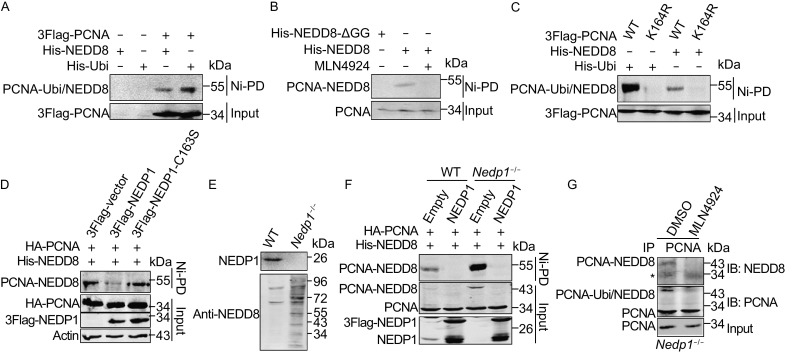
**Modification of PCNA by NEDD8**. (A) Immunoblotting analysis of PCNA NEDDylation in His-tagged NEDD8 conjugates or ubiquitin conjugates enriched by Ni^2+^ pull-down (Ni-PD). (B–D) Analysis of the effect of MLN4924 (B), PCNA-K164R (C), and NEDP1 (D) on PCNA NEDDylation. To obtain the results shown in (A–D), HEK293T cells were transfected with indicated plasmids, and then the whole cell lysates (input) and His-tagged NEDD8 conjugates or ubiquitin conjugates enriched by Ni-PD were subjected to SDS-PAGE and detected by Western blotting with the respective antibodies. (E) Analysis of the protein level of NEDP1 and NEDD8 in *Nedp1* ^−/−^ HEK293T cells. *Nedp1* ^−/−^ HEK293T cells were generated using CRISPR/CAS9 method. NEDP1 and NEDD8 were detected by Western blotting using the indicated antibodies. (F) Immunoblotting analysis of the effect of *Nedp1* knockout on PCNA NEDDylation in His-tagged NEDD8 conjugates. HEK293T WT or *Nedp1* ^−/−^ cells were transfected with indicated plasmids, and then His-tagged NEDD8 conjugates were enriched by Ni-PD and detected by Western blotting with the respective antibodies. (G) Immunoblotting analysis of endogenous PCNA NEDDylation in *Nedp1* ^−/−^ HEK293T cells. *Nedp1* ^−/−^ HEK293T cells were lysed in NP-40 lysis buffer and sonicated for 15 times, then immunoprecipitation was performed using anti-PCNA antibody

The mass spectrum data of NEDD8 substrates screening in the previous study showed that PCNA might be a substrate of NEDD8 (Xirodimas et al., 2008; Coleman et al., 2017). As PCNA is modified by ubiquitin, SUMO and ISG15 (Hoege et al., 2002; Park et al., 2014), we thus assessed whether PCNA was NEDDylated by NEDD8 using Ni^2+^ pull-down assay, in which His-tagged NEDD8 was expressed and NEDD8 conjugates were enriched by Ni^2+^ resin. As shown in Fig. 1A, we identified a specific band similar to PCNA ubiquitination that migrated slower than the unmodified PCNA, representing PCNA NEDDylation. By contrast, we did not observe modified PCNA in cells expressing a mutant NEDD8-ΔGG that lacked the last two-glycine residues (Figs. 1B and S1A). Moreover, we investigated whether NEDD8-activating enzyme inhibitor MLN4924 affected PCNA NEDDylation. As shown in Fig. 1B, MLN4924 abolished PCNA NEDDylation compared to that in untreated cells, indicating that the NEDD8 activating E1 is utilized for PCNA NEDDylation.

As the lysine 164 of PCNA is the modification site for both ubiquitin and SUMO (Bergink and Jentsch, 2009), to investigate whether NEDD8 also targets PCNA at Lys164, we constructed a mutant, PCNA-K164R, and examined its NEDDylation. No band corresponding to NEDDylated PCNA-K164R was detected (Fig. 1C), indicating that PCNA NEDDylation, ubiquitination, and SUMOylation occur on the same lysine residue.

Furthermore, we examined whether NEDP1 acted as the deNEDDylase of PCNA. As shown in Figs. 1D and S1B, NEDP1 significantly inhibited PCNA NEDDylation, but the mutant of NEDP1, NEDP1-C163S, in which the deNEDDylase activity was lost, could not deNEDDylate PCNA. Next, we constructed *Nedp1* ^−/−^ cells and found that NEDD8-conjugated targets were enhanced significantly (Fig. 1E), explaining why only few conjugates were formed by endogenous NEDD8 (due to the presence of NEDP1). Compared to wild type (WT) cells, PCNA NEDDylation also increased significantly in *Nedp1* ^−/−^ cells, which was eliminated by NEDP1 expression (Fig. 1F). These observations suggest that NEDP1 is the specific deNEDDylase for PCNA NEDDylation.

As both PCNA ubiquitination and NEDDylation could not be detected in normal cells, we performed immune precipitation (IP) to detect endogenous NEDDylation of PCNA in *Nedp1* ^−/−^ cells. As shown in Fig. 1G, we succeeded to detect endogenous PCNA NEDDylation, which was abolished by MLN4924 treatment, confirming that PCNA is NEDDylated *in vivo*.

### RAD18 serves as an E3 ligase of PCNA NEDDylation

**Figure 2:**
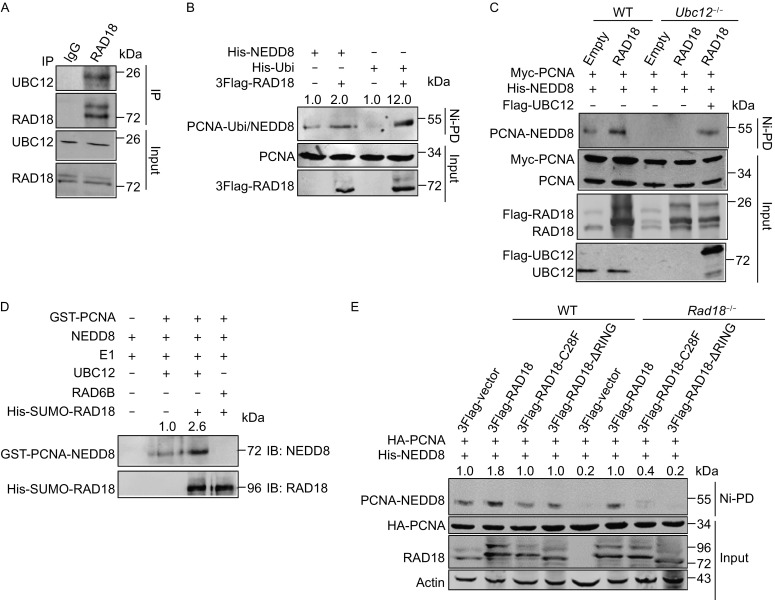
**RAD18 acts as an E3 ligase of PCNA NEDDylation**. (A) Analysis of the interaction between RAD18 and UBC12. HEK293T cells were lysed in NP-40 lysis buffer, and sonicated for 15 times, then immunoprecipitation was performed using antibody against RAD18. (B and C) Immunoblotting analysis of the effect of RAD18 on PCNA NEDDylation in His-tagged NEDD8 conjugates from HEK293T WT (B) or *Ubc12* ^−/−^ cells (C). HEK293T WT cells or *Ubc12* ^−/−^ cells were transfected with the corresponding plasmids, and PCNA NEDDylation or ubiquitination was analyzed by Ni-PD assay and Western blotting with indicated antibodies. (D) Analysis of PCNA NEDDylation by *in vitro* NEDDylation assay. GST-PCNA and His-SUMO-RAD18 were incubated with E1, UBC12 or RAD6B, and NEDD8 at 37°C for 1 h, and then the reactions were terminated by adding SDS buffer and detected by Western blotting with indicated antibody. (E) Immunoblotting analysis of PCNA NEDDylation in *Rad18* ^−/−^ cells. HEK293T WT or *Rad18* ^−/−^ cells were transfected with indicated plasmids, and PCNA NEDDylation was detected as described above. Fold change of PCNA modification intensity was analyzed using odyssey imaging systems (LI-COR, Lincoln, Nebraska USA)

It has been reported that some substrates of NEDD8 and ubiquitin share the same E3 ligase (Enchev et al., 2015), we therefore explored whether RAD18 also functions as the E3 ligase conjugating NEDD8 to PCNA. First, we detected the interaction between RAD18 and UBC12, an E2 of the NEDDylation pathway. Co-IP assay showed that both exogenous and endogenous RAD18 interacted with UBC12 (Figs. 2A and S1C). Next, we examined whether RAD18 promotes PCNA NEDDylation by Ni^2+^ pull-down assay. Indeed, RAD18 strongly enhanced PCNA NEDDylation (Fig. 2B). In addition, we constructed *Ubc12* ^−/−^ HEK293T cell (Fig. S1D) and found that RAD18 could not promote PCNA NEDDylation without UBC12 (Fig. 2C). We further confirmed the effect of RAD18 on PCNA NEDDylation *in vitro*. As shown in Fig. 2D, UBC12/RAD18 but not RAD6B/RAD18 could NEDDylate PCNA, suggesting that RAD18 catalyzes PCNA NEDDylation using NEDD8 specific enzymes *in vivo and vitro*.

The Cys28 in the RING domain of RAD18 is the key site essential for its E3 ligase activity (Huang et al., 2009b), we constructed two RAD18 mutants, C28F and ΔRING, and detected their effects on PCNA NEDDylation. The mutants diminished the activity of RAD18 in mediating PCNA NEDDylation (Fig. 2E, lines 1–4), demonstrating that Cys28 is essential for RAD18 to catalyze NEDDylation pathway. To investigate whether PCNA NEDDylation is dependent on RAD18, we constructed a *Rad18*-deficient stable HEK293T cell line using the Fast TALE^TM^ TALEN Assembly Kit and confirmed *Rad18* knockout by Western blotting using an antibody against RAD18 (Fig. S1E). Then we examined the level of PCNA NEDDylation in *Rad18* ^−/−^ cells. With *Rad18* depletion, PCNA NEDDylation was abolished totally compared to that in WT cells, and only WT RAD18 but not the mutants could recover PCNA NEDDylation (Fig. 2E, lines 5–8). In summary, these data indicate that RAD18 acts as an E3 ligase of PCNA NEDDylation, which depends on UBC12.

### NEDDylated PCNA accumulates under H_2_O_2_-induced oxidative stress

**Figure 3:**
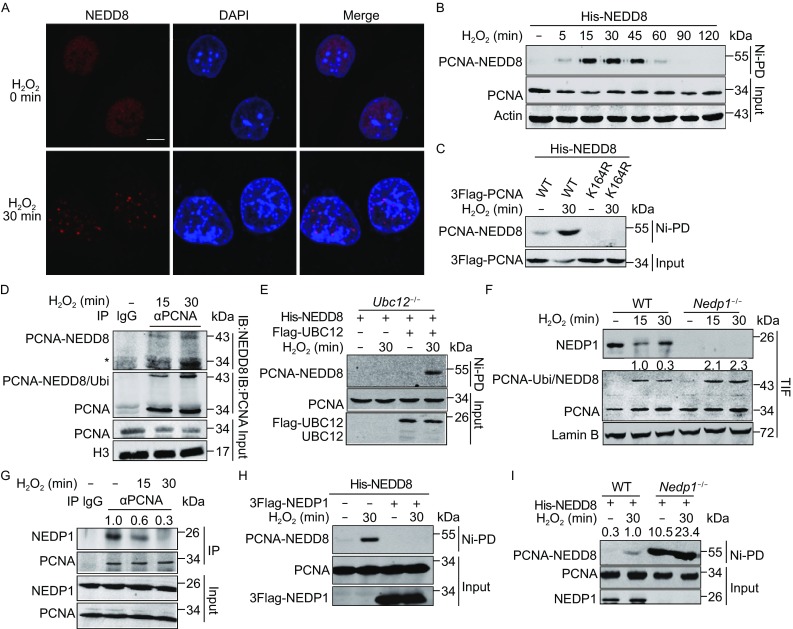
**Hydrogen peroxide promotes PCNA NEDDylation**. (A) Immunofluorescence analysis of NEDD8 localization after H_2_O_2_ treatment. HeLa cells were treated with or without 800 μmol/L H_2_O_2_ for 30 min, and then endogenous NEDD8 was examined by immunofluorescence staining using anti-NEDD8 antibody. The scale bar is 10 μm. (B) A time course analysis of PCNA NEDDylation in His-tagged NEDD8 conjugates enriched by Ni-PD in response to H_2_O_2_-induced oxidative stress. In (B), (C), and (E), HEK293T WT or *Ubc12* ^−/−^ cells were transfected with the indicated plasmids for 36 h and harvested after treatment with 800 μmol/L H_2_O_2_ for the indicated time. The NEDD8 conjugates were enriched through Ni-PD and detected by Western blotting using anti-PCNA antibody. (C) Analysis of the effect of K164R on PCNA NEDDylation. (D) Analysis of endogenous PCNA NEDDylation in response to H_2_O_2_-induced oxidative stress. HEK293T cells treated with 800 μmol/L H_2_O_2_ for the indicated time were lysed in NP-40 lysis buffer and sonicated for 15 times. Immunoprecipitation was performed using anti-PCNA antibody. (E) Immunoblotting analysis of the effect of *Ubc12* knockout on PCNA NEDDylation in His-tagged NEDD8 conjugates. (F) Analysis of the abundance of NEDP1 and PCNA modification in the Trixon-X100 insoluble fraction (TIF) in HEK293T WT or *Nedp1* ^−/−^ cells under oxidative stress. HEK293T WT or *Nedp1* ^−/−^ cells were treated with 800 μmol/L H_2_O_2_ for the indicated time. Then the TIF was isolated and subjected to Western blotting analysis with anti-PCNA and anti-NEDP1 antibodies. Fold change of PCNA modification intensity was analyzed using odyssey imaging systems. (G) Analysis of the interaction between PCNA and NEDP1 in response to H_2_O_2_ treatment. HEK293T cells were treated with H_2_O_2_ for indicated time, and then lysed in Triton-X100 lysis buffer and sonicated for 15 times. Immunoprecipitation was performed using antibody against PCNA. (H and I) Analysis of the effect of NEDP1 expression (H) or *Nedp1* knockout (I) on accumulation of NEDDylated PCNA in response to H_2_O_2_ treatment. HEK293T WT or *Nedp1* ^−/−^ cells were transfected with His-NEDD8, and then the cells were treated with or without 800 μmol/L H_2_O_2_ for 30 min before NEDDylated PCNA was enriched by Ni-PD and detected by Western blotting using anti-PCNA antibody

Previous research revealed that PCNA is monoubiquitinated under H_2_O_2_-induced oxidative stress, and monoubiquitinated PCNA helps recruiting polη to bypass DNA lesions (Zlatanou et al., 2011). We first detected whether NEDD8 accumulated at DNA damage sites after H_2_O_2_ treatment. As shown in Fig. 3A, NEDD8 formed foci in HeLa cells treated with H_2_O_2_, suggesting that NEDD8 participates in the H_2_O_2_-induced DDR pathway. To further assess whether PCNA NEDDylation also involves in the DDR pathway, we treated cells with 800 µmol/L H_2_O_2_ for various times and performed Ni^2+^ pull-down assay to examine PCNA NEDDylation. As shown in Fig. 3B, NEDDylation of PCNA occurred at 5 min, reached a peak at 15 min, decreased at 60 min, and finally disappeared after 90 min of H_2_O_2_ treatment. Meanwhile, in cells with PCNA-K164R, accumulation of NEDDylated PCNA did not occur after H_2_O_2_ treatment (Fig. 3C), indicating that the increased NEDDylation of PCNA is dependent on the conserved modification residue Lys164. We also observed an increase of endogenous PCNA NEDDylation in response to H_2_O_2_ treatment (Fig. 3D). In addition, NEDDylated PCNA also accumulated under DNA damage induced by UV irradiation (Fig. S2A and S2D). These data strongly suggest that PCNA NEDDylation is involved in the DDR pathway induced by various stimulations.

Previous study reported that oxidative stress causes a general increase in NEDDylation through ubiquitin E1 enzyme UBE1 but independent of the NEDD8 E1 enzyme (Leidecker et al., 2012), we questioned whether the increase in PCNA NEDDylation required the NEDD8 E1 APPBP1-UBA3. As shown in Fig. S2C, in MLN4924-treated 293T cells, we performed Ni^2+^ pull-down assay and observed a significant inhibition of MLN4924 on PCNA NEDDylation but not its ubiqitination, suggesting that PCNA NEDDylation utilizes the NEDD8 E1 under oxidative stress. In addition, UBC12-C111S, an activity-deficient mutant of UBC12 and a dominant blocker of the NEDDylation pathway by reducing the pool of free NEDD8, also impeded the increase in PCNA NEDDylation (Fig. S2B). Moreover, in *Ubc12* ^−/−^ cells, we did not detect the increase of PCNA NEDDylation, while expression of UBC12 recovered the accumulation of NEDDylated PCNA (Fig. 3E).

Next, we investigated whether NEDP1 involved in the H_2_O_2_ treatment-induced DNA damage response. As the modified PCNA accumulated on chromatin during S-phase and became resistant to Triton-X100 extraction (Kannouche et al., 2004), we treated HEK293T cells with Triton-X100 and then detected whether NEDP1 affected PCNA modification. Consistent with the reduction of NEDP1 in the Triton-X100 insoluble fraction (TIF), we observed an obvious increase in modified PCNA (Fig. 3F, lanes 2–3 vs. lane 1), and in *Nedp1* ^−/−^ cells, modified PCNA increased significantly compared to WT cells under oxidative stress (Fig. 3F, lanes 5–6 vs. lanes 2–3). Meanwhile, the result of co-IP showed that the interaction between PCNA and NEDP1 decreased after H_2_O_2_ treatment (Fig. 3G). Moreover, expression of NEDP1 abolished PCNA NEDDylation and inhibited NEDDylated PCNA under oxidative stress (Fig. 3H), but *Nedp1* knockout enhanced PCNA NEDDylation significantly (Fig. 3I). These findings indicate that NEDP1 is involved in the DDR, and the increase in PCNA NEDDylation under H_2_O_2_-induced oxidative stress is associated with dissociation of NEDP1 from PCNA. Together, these results demonstrate that PCNA NEDDylation participates in oxidative stress-induced DDR.

### RAD18 is essential for the accumulation of NEDDylated PCNA under oxidative stress

**Figure 4:**
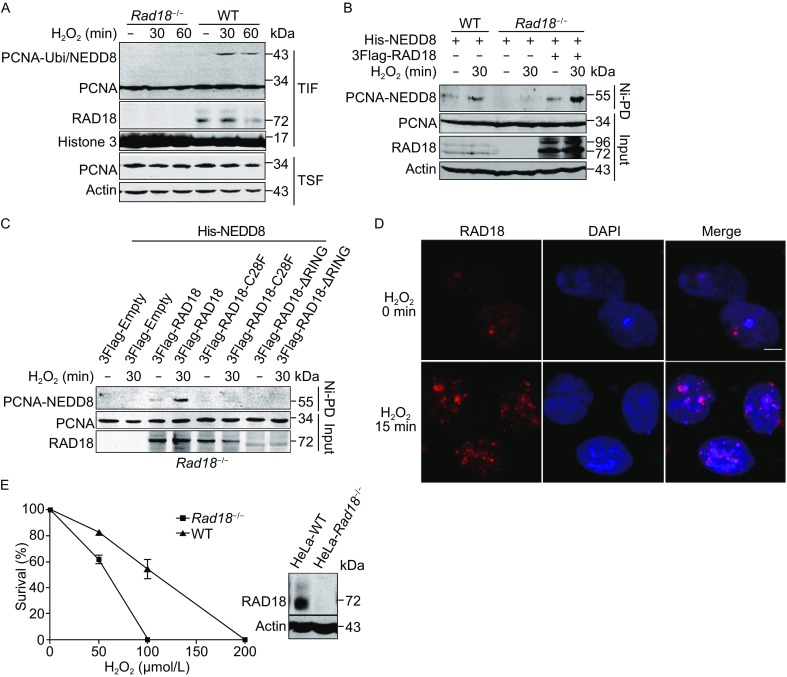
**RAD18 is essential for the accumulation of NEDDylated PCNA under oxidative stress**. (A) Immunoblotting analysis of the effect of RAD18 on oxidative stress-induced PCNA ubiquitination and NEDDylation in the TIF from WT or *Rad18* ^−/−^ HEK293T cells. Cells were treated with 800 μmol/L H_2_O_2_ for the indicated time, and then the TIF was isolated and subjected to staining with anti-PCNA antibody. (B and C) Immunoblotting analysis of the effect of RAD18 on PCNA NEDDylation under H_2_O_2_-induced oxidative stress. WT and *Rad18* ^−/−^ HEK293T cells were transfected with the indicated plasmids, and at 36 h after transfection, the cells were treated with or without 800 μmol/L H_2_O_2_ for 30 min before PCNA NEDDylation was detected as described above. (D) Immunofluorescence analysis of RAD18 localization after H_2_O_2_ treatment. HeLa cells were treated with or without 800 μmol/L H_2_O_2_ for 30 min, and then endogenous RAD18 was examined by immunofluorescence staining using anti-RAD18 antibody. The scale bar is 10 μm. (E) Analysis of the effect of RAD18 on cell survival against oxidative stress-induced DNA damage. HeLa WT and *Rad18* ^−/−^ cells were cultured and treated with the indicated doses of H_2_O_2_, and then the cells were cultured for 12 days before colony counting. The error bars represented the standard error of the mean (SEM). Anti-RAD18 antibody was used for Western blotting analysis

Because RAD18 acted as an E3 ligase of PCNA NEDDylation, we questioned whether oxidative stress-induced accumulation of NEDDylated PCNA is dependent on RAD18. In H_2_O_2_ treated WT cells, we observed an obvious increase in modified PCNA in the TIF, while in *Rad18* ^−/−^ cells, we did not detect modified bands of PCNA under oxidative stress (Fig. 4A). As we could not directly distinguish PCNA NEDDylation from ubiquitination in Fig. 4A, we thus performed Ni^2+^ pull-down assay to detect NEDDylated PCNA. As shown in Fig. 4B, no NEDDylated PCNA could be detected in *Rad18* ^−/−^ cells, even with H_2_O_2_ treatment. In addition, WT RAD18 but not the inactive enzyme mutants of RAD18, C28F or ΔRING could rescue PCNA NEDDylation (Fig. 4B and 4C). These data indicate that RAD18 is required for PCNA NEDDylation to participate in the DDR pathway.

As RAD18 was essential in the cellular response to UV irradiation-induced stress (Watanabe et al., 2004), we next investigated whether RAD18 accumulated at DNA damage sites induced by H_2_O_2_. As shown in Fig. 4D, RAD18 formed foci in HeLa cells treated with H_2_O_2_, suggesting that RAD18 indeed participates in the H_2_O_2_-induced DDR pathway. To further investigate whether RAD18 is required for cell survival under H_2_O_2_-induced oxidative stress, we performed colony-forming assay and found that the survival ability of *Rad18*-deficient cells under oxidative stress reduced obviously (Fig. 4E). This finding suggests that RAD18 is essential for the cellular response to H_2_O_2_-induced oxidative stress and PCNA NEDDylation may play an important role in this process.

### NEDDylation antagonizes PCNA ubiquitination in response to oxidative DNA damage

**Figure 5:**
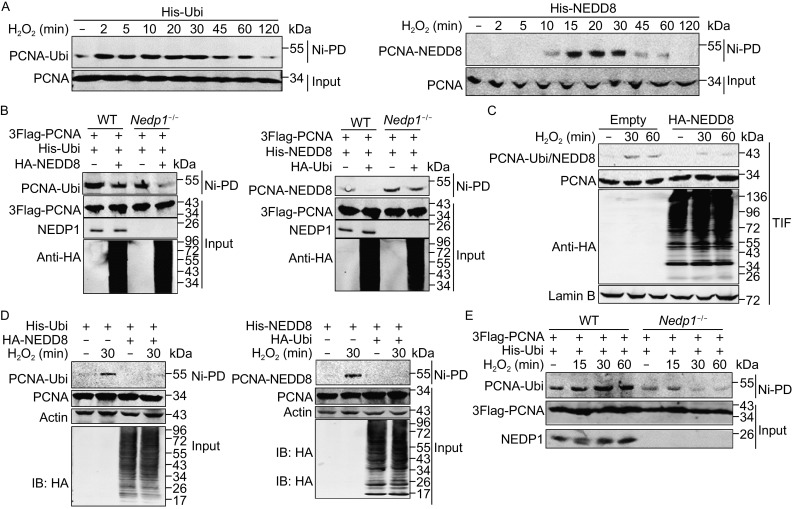
**NEDDylation regulates oxidative DNA damage-induced PCNA ubiquitination**. (A) A time course analysis of NEDDylated PCNA and ubiquitinated PCNA in response to H_2_O_2_ treatment. HEK293T cells were transfected with indicated plasmids for 36 h and treated with or without 800 μmol/L H_2_O_2_ for different time. And then PCNA ubiquitination and NEDDylation were analyzed by Ni-PD and Western blotting with anti-PCNA antibody. (B) Immunoblotting analysis of the effect of NEDD8 expression and *Nedp1* deletion on PCNA ubiquitination. HEK293T WT or *Nedp1* ^−/−^ cells were transfected with the indicated plasmids for 36 h and then Ni-PD assay was performed to analyze PCNA ubiquitination and NEDDylation. (C) Analysis of the effect of NEDD8 expression on the accumulation of modified PCNA in TIF. Empty vector or HA-NEDD8 was transfected into HEK293T cells, and then the TIF was separated and analyzed as described above. (D) Analysis of the effect of NEDD8 on PCNA ubiquitination in response to H_2_O_2_ treatment. In (D) and (E), HEK293T WT or *Nedp1* ^−/−^ cells were transfected with indicated plasmids for 36 h and then treated with or without 800 μmol/L H_2_O_2_ for different time. Then PCNA ubiquitination and NEDDylation were analyzed as described above. (E) Analysis of the effect of *Nedp1* deletion on accumulation of ubiquitinated PCNA in response to H_2_O_2_ treatment

Because oxidative stress induces both PCNA NEDDylation and ubiquitination, we sought to clarify the relationship between these two modifications. First, we detected whether the change of PCNA-NEDD8 signal was different from that of ubiquitinated PCNA in response to DNA damage. As shown in Fig. 5A, PCNA ubiquitination increased significantly and reached a peak at 2 min, whereas PCNA NEDDylation got to the peak at 15 min, later than its ubiquitination (Figs. 3A, 5A, right panel). In UV-treated cells, we also found that PCNA ubiquitination reached a peak at 6 h after UV treatment, but decreased at 12 h, while its NEDDylation got to the peak at 12 h and lasted for 24 h (Fig. S2D). As PCNA NEDDylation and ubiquitination shared the same modified lysine K164, we want to know whether PCNA NEDDylation and ubiquitination compete with each other. As shown in Fig. 5B, expression of NEDD8 inhibited PCNA ubiquitination (line 2 vs. line 1, left panel), and *vice versa* (line 2 vs. line 1, right panel), suggesting that PCNA NEDDylation antagonizes its ubiquitination. In addition, in *Nedp1* ^−/−^ cells, we examined whether *Nedp1* knockout affected PCNA ubiquitination. As shown in Fig. 5B, PCNA ubiquitination decreased (line 3 vs. line 1, left panel) but its NEDDylation increased (line 3 vs. line 1, right panel) in *Nedp1* ^−/−^ cells, and exogenous NEDD8 inhibited PCNA ubiquitination (line 4 vs. line 3, left panel), and *vice versa* (line 4 vs. line 3, right panel). Moreover, in *Ubc12* ^−/−^ cells, we found that PCNA ubiquitination increased, which was reduced by UBC12 re-expression (Fig. S3A). This data also indicates that NEDD8 antagonizes PCNA ubiquitination.

To investigate the effect of NEDDylation on PCNA ubiquitination in response to DNA damage, we treated cells with H_2_O_2_ and performed chromatin fractionation to detect PCNA modification. We found that NEDD8 blocked the increase of PCNA modification in the TIF (Fig. 5C). Furthermore, Ni^2+^ pull-down assay also showed that NEDD8 antagonized the accumulation of ubiquitinated PCNA (Fig. 5D, left panel), and *vice versa* (Fig. 5D, right panel). Moreover, we assessed whether *Nedp1* knockout and *Ubc12* knockout affected PCNA ubiquitination induced by H_2_O_2_ treatment. As shown in Fig. 5E, compared to WT cells, the accumulation of ubiquitinated PCNA was significantly inhibited in *Nedp1* ^−/−^ cells. And in *Ubc12* ^−/−^ cells, PCNA ubiquitination increased more significantly, while UBC12 re-expression reduced this increase (Fig. S3B). These results indicate that enhanced NEDDylation antagonizes PCNA ubiquitination in response to oxidative stress.

USP1 is a deubiquitinase of PCNA, which decreases under UV-induced stress (Huang et al., 2006). Here, we also examined whether USP1 affected PCNA NEDDylation. As shown in Fig. S4A, USP1 inhibited PCNA ubiquitination but not affected PCNA NEDDylation. In addition, in response to H_2_O_2_ treatment, we found that USP1 reduced the accumulation of ubiquitinated PCNA (Fig. S4B) but not inhibited NEDDylated PCNA (Fig. S4C), suggesting that USP1 does not affect PCNA NEDDylation.

### NEDDylation pathway regulates polη recruitment

**Figure 6:**
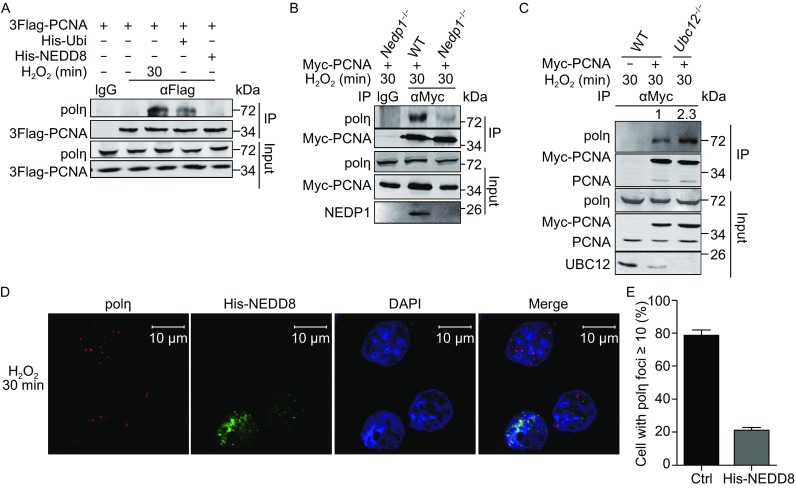
**NEDDylation affects polη recruitment under oxidative stress**. (A) Analysis of the effect of ubiquitin and NEDD8 on PCNA-polη interaction in response to H_2_O_2_ treatment. In (A–C), HEK293T WT, *Nedp1* ^−/−^ or *Ubc12* ^−/−^ cells were transfected with the indicated plasmids. At 36 h after transfection, cells were treated with 800 μmol/L H_2_O_2_ for the indicated time, and then cells were lysed in Triton-X100 lysis buffer and sonicated for 15 times. Co-IP was performed using antibody against anti-Flag or anti-Myc antibody. (B and C) Analysis of the effect of *Nedp1* knockout (B) or *Ubc12* knockout (C) on PCNA-polη interaction in response to H_2_O_2_ treatment. Fold change of PCNA modification intensity was analyzed using odyssey imaging systems. (D) Immunofluorescence analysis of the effect of NEDD8 on H_2_O_2_-induced polη foci. HeLa cells were transfected with His-NEDD8 for 24 h, followed by 800 μmol/L H_2_O_2_ treatment for 30 min. Then immunofluorescence microscopy was performed to analyze the formation of polη foci. The scale bar is 10 μm. (E) Quantitative analysis of polη foci under oxidative stress. Cells (100 in total) with polη foci ≥10 were counted, and quantitative results are provided as mean ± SEM

As NEDD8 antagonizes PCNA ubiquitination, we wondered whether promotion of NEDDylation affects PCNA-polη interaction. Notably, immunoprecipitation of PCNA by anti-Flag antibody enriched polη under oxidative stress (Fig. 6A, lane 3). Moreover, in cells not treated with H_2_O_2_, expression of ubiquitin but not NEDD8 promoted the recruitment of polη to PCNA (Fig. 6A, lanes 4 and 5). These results indicate that PCNA ubiquitination is required for the recruitment of polη and PCNA NEDDylation does not directly promote this recruitment. Next, we examined the PCNA-polη interaction in *Nedp1* ^−/−^ cells and *Ubc12* ^−/−^ cells, and detected the effect of NEDD8 on polη foci in response to H_2_O_2_ treatment. As shown in Fig. 6B, *Nedp1* knockout reduced the interaction between PCNA and polη, suggesting that enhanced NEDDylation reduced PCNA ubiquitination, thus impeded polη recruitment. However, in *Ubc12* ^−/−^ cells, PCNA-polη interaction increased (Fig. 6C), indicating that loss of PCNA NEDDylation promotes polη recruitment. In addition, Immunofluorescence assay showed that NEDD8 expression blocked polη foci formation under oxidative stress (Fig. 6D and 6E). Together, these results demonstrate that up-regulation of NEDDylation by NEDD8 overexpression or *Nedp1* knockout antagonizes PCNA ubiquitination and thus inhibits polη recruitment, while *Ubc12* knockout promotes PCNA ubiquitination and enhances polη recruitment.

### NEDDylation regulates cell sensitivity to H_2_O_2_-induced oxidative stress

**Figure 7:**
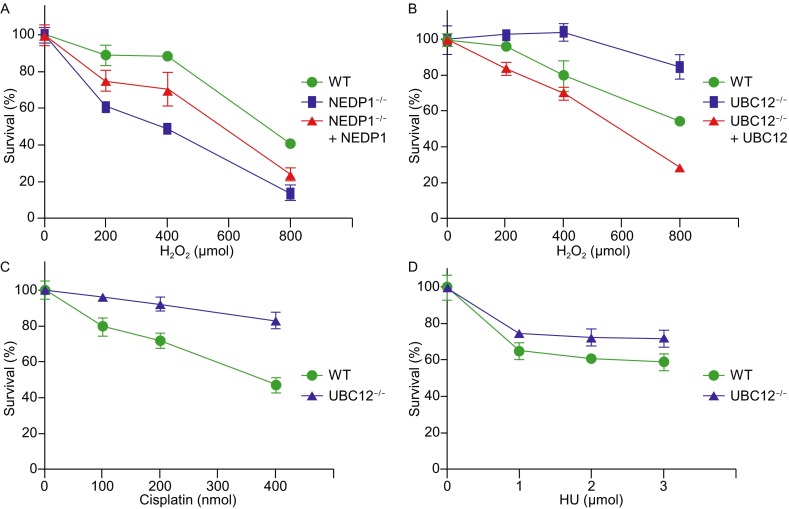
**NEDDylation regulates cell sensitivity to H**_**2**_ **O**_**2**_ **-induced oxidative stress**. (A and B) Analysis of the effect of NEDP1 deletion (A) or UBC12 deletion on cell sensitivity in response to H_2_O_2_ treatment. In (A and B), HEK293T WT, *Nedp1* ^−/−^ or *Ubc12* ^−/−^ cells were cultured in 96-well plates. After 24 h, cells were treated with H_2_O_2_ at indicated dose for 1 h, and then incubated with CCK8 for 4 h. OD_450_ was measured using BioTek Cytation 5, triplicates were performed. Results are provided as mean ± SD. (C and D) Analysis of the effect of UBC12 deletion on cell sensitivity in response to cisplatin (C) or HU (D) treatment. In (C and D), HEK293T WT or *Ubc12* ^−/−^ cells were cultured and cell cytotoxicity assay was performed as described above

As polη-mediated TLS is essential for genome integrity maintenance and cell survival under UV treatment (Han et al., 2014), we further detected whether NEDDylation regulated cell sensitivity to H_2_O_2_ treatment. As shown in Fig. 7A, HEK293T WT or *Nedp1* ^−/−^ cells were treated with H_2_O_2_ at different doses, followed by incubating with CCK8 (cell counting kit 8) and then cell density was examined. In comparison to WT cells, *Nedp1* ^−/−^ cells were less resistant against H_2_O_2_ treatment, while NEDP1 re-expression increased cell survival (Fig. 7A). This result indicates that enhanced NEDDylation increases cell sensitivity to H_2_O_2_ treatment.

While in *Ubc12* ^−/−^ cells, we found that UBC12 deletion increased cell survival while UBC12 re-expression increased cell sensitivity to oxidative stress induced by H_2_O_2_ treatment (Fig. 7B). In addition, *Ubc12* ^−/−^ cells were more resistant against cisplatin or HU (hydroxyurea) treatment in comparison to WT cells (Fig. 7C and 7D). These data indicate that in *Ubc12* ^−/−^ cells, impaired NEDDylation increases cell survival in response to DNA damaging agents.

## DISCUSSION

**Figure 8:**
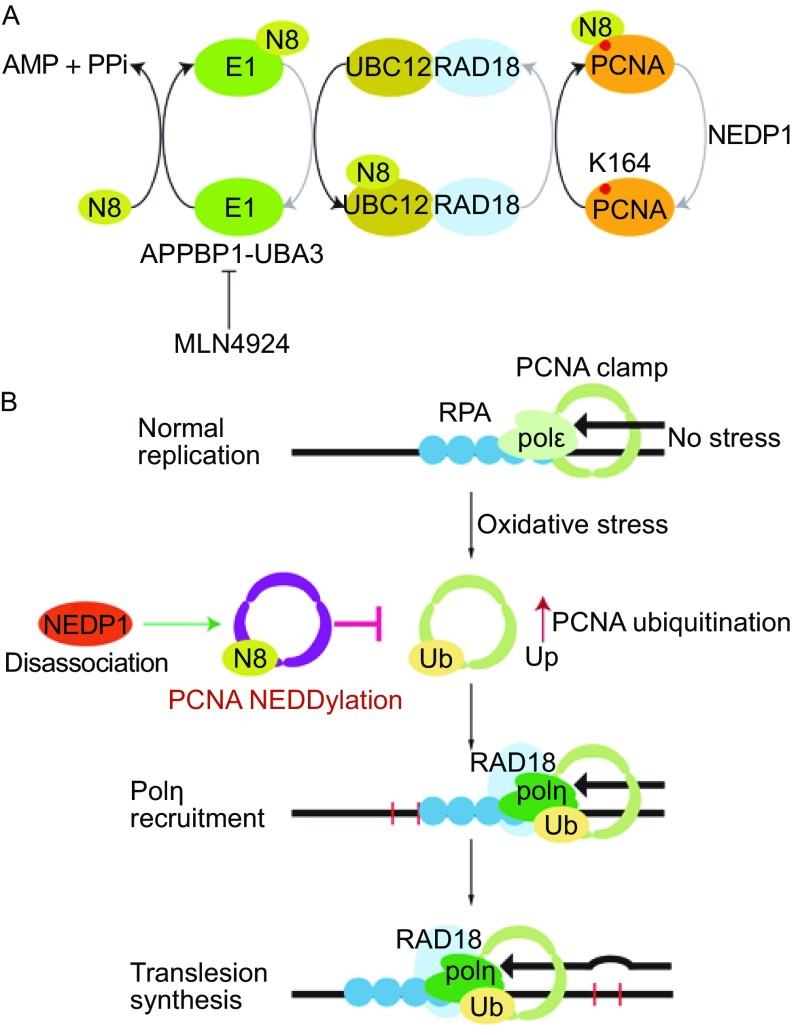
**Model of PCNA NEDDylation**. (A) Illustration of PCNA NEDDylation cascade. In an ATP-dependent manner, a NEDD8-adenylate intermediate is produced to form a NEDD8-E1 (APPBP1-UBA3) complex. Then NEDD8 is transferred from the E1 to the E2 (UBC12), which combines with the E3 ligase RAD18. NEDD8 is subsequently conjugated to the Lys164 of PCNA. In this process, MLN4924 can inhibit the function of E1, thus impeding PCNA NEDDylation, and NEDP1 acts as the deNEDDylase of PCNA. (B) The molecular function of PCNA NEDDylation in regulating its monoubiquitination and TLS. In cells without stress, PCNA acts as a clamp to recruiting polε for the normal replication process. When cells were under oxidative DNA damage, monoubiquitinated PCNA recruits TLS polymerase polη to bypass the lesions. Meanwhile, NEDP1 disassociates from PCNA and PCNA is NEDDylated. PCNA NEDDylation antagonizes its monoubiquitination and leads to defective PCNA-polη interaction and reduced TLS activity

In our study, we identify an important role for NEDD8 in regulating polη recruitment in the DDR pathway. As shown in Fig. 8A, we demonstrate that PCNA is modified by NEDD8 at the conserved Lys164. PCNA NEDDylation utilizes the conserved NEDD8 system, which is catalyzed by an E3 ligase RAD18. NEDP1 acts as the deNEDDylase of PCNA, and in response to oxidative damage, NEDP1 disassociates from PCNA and results in the accumulation of NEDDylated PCNA (Fig. 8B). More importantly, *Ubc12* knockout promotes PCNA ubiquitination while *Nedp1* knockout or NEDD8 expression inhibits PCNA ubiquitination and further reduces polη recruitment.

Recent studies have revealed that PCNA is targeted by ubiquitin and SUMO at the same Lys164, and the ubiquitination and SUMOylation of PCNA induced by DNA damage promotes different branches of DNA damage bypass (Hoege et al., 2002; Papouli et al., 2005). Interestingly, our results show that PCNA NEDDylation also occurs at Lys164 and utilizes the same E3 ligase RAD18, suggesting a close relationship between PCNA ubiquitination and NEDDylation. Thus, it is interesting to clarify the functional correlation between these two posttranslational modifications of PCNA. Interestingly, in response to H_2_O_2_ treatment, we found that the peak of NEDDylated PCNA occurred later than that of ubiquitinated PCNA. Moreover, NEDD8 expression inhibits PCNA ubiquitination, and increased PCNA NEDDylation in *Nedp1* ^−/−^ cells suppressed its ubiquitination, but impaired PCNA NEDDylation in *Ubc12* ^−/−^ cells promoted its ubiquitination (Figs. 5 and S3). These data indicate that PCNA NEDDylation antagonizes its monoubiquitination in response to DNA damage and provides a new regulatory mechanism for the involvement of PCNA ubiquitination in the process of TLS. As RAD18 catalyzes both PCNA NEDDylation and ubiquitination, it is important to explain how RAD18 could recognize ubiquitin and NEDD8. Our results show that RAD18 interacts with UBC12 and catalyzes PCNA NEDDylation in a UBC12-dependent manner (Fig. 2). Meanwhile, RAD18 also forms a complex with RAD6B, an E2 of ubiquitin, to mono-ubiquitinate PCNA (Bailly et al., 1994). These observations suggest that RAD18 catalyzes both NEDDylation and ubiquitination of PCNA through interacting with their respective E2s.

It is well known that the monoubiquitination of PCNA plays a key role in the regulation of TLS. In response to DNA damage, PCNA is monoubiquitinated by the RAD18-RAD6 complex, which provides a platform for recruiting certain repair factors and specific translesion DNA polymerase such as polη (Hoege et al., 2002; Kannouche et al., 2004). The specialized TLS polymerases then bypass the DNA damage sites without disturbing the continuity of the replication fork but results in a high level of mutagenesis. Our data revealed that PCNA NEDDylation occurred later than its ubiquitination, ubiquitin but not NEDD8 could promote the interaction between PCNA and polη. In addition, *Ubc12* knockout enhanced PCNA-polη interaction, but NEDD8 expression or NEDP1 deletion reduced the recruitment of polη (Fig. 6), indicating that enhanced NEDDylation reduces excessive polη staying on the replication fork to avoid high rate of mutagenesis. We therefore conclude that under H_2_O_2_ or UV-induced stress, PCNA NEDDylation occurs and antagonizes PCNA monoubiquitination and controls polη recruitment to avoid the excessive error-prone bypass.

Furthermore, the discovery of the detailed mechanism underlying how NEDP1 regulates TLS is important. In response to H_2_O_2_-induced oxidative stress, we found that NEDP1 disassociated from PCNA, consistent with the increase of PCNA NEDDylation (Fig. 3F and 3G). In addition, in the *Nedp1* ^−/−^ cells, NEDD8-conjugates including NEDDylated PCNA increased obviously, but PCNA ubiquitination reduced significantly (Fig. 5B). This result indicates that the dissociation of NEDP1 from PCNA contributes to the increase of NEDDylated PCNA. Moreover, NEDP1 deletion reduced PCNA-polη interaction, thus prevented TLS pathway and increased cell sensitivity to H_2_O_2_-induced oxidative stress (Figs. 6B and 7A). Together, we demonstrate that NEDP1 regulates TLS and cell survival through regulating PCNA NEDDylation and its ubiquitination.

As both the increased ratio of free NEDD8 over ubiquitin and oxidative stress induce global atypical NEDDylation using ubiquitin enzyme UBE1, which could transfer NEDD8 to RAD6B (also called UBE2B), the E2 of PCNA ubiquitination (Hjerpe et al., 2012; Leidecker et al., 2012), it is possible that the ubiquitin enzymes cascade UBE1-RAD6B-RAD18 can also be utilized to mediate oxidative stress-induced PCNA NEDDylation. Our *in vivo* and *in vitro* NEDDylation assay showed that UBC12 is an E2 of PCNA NEDDylation (Fig. 2C and 2D) and MLN4924 could inhibit PCNA NEDDylation significantly when NEDD8 was overexpressed or under oxidative stress (Figs. 1B and S2C); therefore, NEDD8 should utilize common NEDD8 enzymes to target PCNA. Consideration of the criteria for identifing NEDD8 substrates (Enchev et al., 2015), in future, more experiments such as constructing cells with endogenously expressed His-tagged NEDD8 using CRISPR-CAS9 technology should be performed to validate the endogenous NEDDylation and investigate the functional significance of PCNA NEDDylation.

As mutation and inactivation of polη cause xeroderma pigmentosum variant (XPV), a type of autosomal recessive disease, which is prone to developing malignant skin cancer (Johnson et al., 1999; Han et al., 2014), we evaluated the clinical relevance of NEDDylation in skin cancer by bioinformatics analysis using SKCM (Skin Cutaneous Melanoma) dataset from GEPIA (gene expression profiling interactive analysis) (http://gepia.cancer-pku.cn/). As shown in Fig. S5A, the expression of polη was significantly higher in SKCM tissues in comparison to that of normal tissues. Meanwhile, correlative analysis revealed that low level of polη is associated with higher survival of SKCM patients (Fig. S5B), indicating that the highly expressed polη is associated with SKCM. In addition, we found that UBC12 was also expressed highly in the SKCM samples (Fig. S5C), suggesting that defective TLS activity caused by abnormal NEDDylation is also correlated with SKCM. In addition, in response to H_2_O_2_ or cisplatin treatment, as UBC12 deletion reduced cell sensitivity while UBC12 re-expression reduced cell survival (Fig. 7B and 7C), it is possible that enhanced NEDDylation of PCNA could reduce TLS activity and the sensitivity of tumor cells to cisplatin treatment, which needs further study in the future.

In summary, we identify PCNA as a new substrate of NEDD8 that has an important role in the DDR pathway. In response to oxidative DNA damage, NEDDylation regulates polη recruitment and cell survival by antagonizing PCNA ubiquitination. Overall, the present study provides new insights into the mechanism through which NEDDylation regulates the oxidative stress-induced DDR.

## MATERIALS AND METHODS

### Cell culture and transfection

HEK293T and HeLa cells (ATCC) were grown in Dulbecco’s Modified Eagle medium (Gibco, Carlsbad, CA, USA) supplemented with 10% fetal bovine serum in 5% CO_2_ at 37°C, and after 24 h in culture, cells were transfected with the indicated plasmids using the transfection reagent PEI (Polyscience, Warrington, PA, USA) following the manufacturer’s protocol.

### Plasmids constructions and antibodies

3Flag-PCNA, 3Flag-PCNA-K164R, 3Flag-RAD18, 3Flag-RAD18-C28F, 3Flag-RAD18-ΔRING, 3Flag-NEDP1, and 3Flag-NEDP1-C163S were cloned into the pCDNA-3Flag vector through restriction sites *Eco*RI and *Xho*I. HA-PCNA was constructed by PCR and cloned into the pRK-HA vector. 3HA-polη was cloned into the pCMV-3HA vector. To construct His-Ubiquitin, His-NEDD8 and His-PCNA, the full-length cDNAs of ubiquitin, NEDD8 and PCNA were digested with *Eco*RI and *Not*I and inserted into the pEF1-c-6His vector. Myc-PCNA and Myc-UBC12 were cloned into pCDNA-Myc vector.

PCNA mouse monoclonal antibody (BE0029) and histone H3 monoclonal antibody (BE3015) were purchased from EASYBIO (Seoul, Korea); RAD18 rabbit monoclonal antibody (D2B8) was purchased from CST (Danvers, MA, USA); polη rabbit monoclonal antibody (BS6695) was purchased from Bioworld (St. Louis Park, MN, USA); NEDD8 rabbit monoclonal antibody (Y297) was purchased from Epitomics (Cambridge, MA, USA); HA mouse monoclonal antibody (H9658) and Flag mouse monoclonal antibody (F3165) were purchased from Sigma-Aldrich (St. Louis, MO, USA); His mouse monoclonal antibody (D291-3) and actin rabbit monoclonal antibody (PM053) were purchased from MBL (Nagano, Japan); and NEDP1 monoclonal antibody (F1512) was purchased from Santa Cruz Biotechnology (Dallas, TX, USA).

### *Nedp1* and *Ubc12* knockout cell line

The *Nedp1* ^−/−^ and *Ubc12* ^−/−^ cell line were generated using the CRISPR/CAS9 method. The sgRNA sequences targeting NEDP1 were tacatggacagtctactg-NGG and gccatgttccttgaaccac-NGG. The sgRNA sequences targeting UBC12 were gagtcggcgggcggcaccaa-NGG and ctgcggatccagaagggtag-NGG. HEK293T cells were transfected with sgRNA vector and CAS9 vector, 48 h later, cells expressing sgRNAs were divided by FACS for green fluorescence and single cells were plated in a 96-well dish to screen for positive monoclonal cells.

### *Rad18* knockout cell line

The *Rad18* ^−/−^ cell line was generated using the transcription activator-like effector nuclease (TALEN) method. The Fast TALE^TM^ TALEN Assembly Kit (SIDANSAI, Shanghai, China) was used to construct TALEN vectors targeting RAD18 by one-step ligation. The target sequences were L-ttcttgatcagagaaat and R-tttcttttatcaacaact. HEK293T cells were transfected with TALEN vectors and selected in the presence of puromycin for 3 days until all control cells transfected with empty vectors had died. The remained cells were diluted and plated in a 96-well dish to screen for positive monoclonal cells.

### Ni^2+^ Pull-down assay (Ni-PD)

His-tagged NEDD8 conjugates and ubiquitin conjugates were maintained as previously described in denaturing conditions (Li et al., 2014). HEK293T cells transfected with the indicated plasmids were lysed in 6 mL lysis buffer for 30 min and then incubated with 80 μL Ni^2+^ resin for 4 h. The lysates were washed with buffers 1, 2, 3, and 4 in turn, and then the modified proteins were eluted in 30 μL elution buffer and boiled in 30 μL 2× SDS loading buffer. The samples were subjected to Western blotting analysis.

### Subcellular fractionation and immunoprecipitation

HEK293T cells were cultured in 10-cm dishes and transfected with the indicated plasmids. Cells were harvested in 10 mL pre-cooled PBS and lysed for 30 min in 0.2% Triton lysis buffer with 5 μL cocktail (1 mmol/L phenylmethylsulfonyl fluoride, 1 μg/mL leupeptin, 1 μg/mL aprotinin, and 1 μg/mL pepstatin). Then the lysates were isolated as the Triton-soluble fraction, and the pellets were resuspended in 2× SDS loading buffer as the Triton-insoluble fraction or in buffer appropriate for other assays.

For immunoprecipitation assays, cells were lysed in 0.2% Triton lysis buffer, and the resulting pellets were resuspended in NP-40 lysis buffer with 0.1% SDS before being subjected to sonication fifteen times (5 s each) at 200 W. Next, the lysates were incubated with the indicated antibodies overnight at 4°C and precipitated by protein G for 5 h. After three washes with NP-40 lysis buffer, the samples were boiled with 2× SDS loading buffer and subjected to SDS-polyacrylamide gel electrophoresis (PAGE) and Western blotting analysis.

### *In vitro* NEDDylation assay

*In vitro* NEDDylation analysis was performed using the NEDDylation kit (Enzo, Farmingdale, NY, USA). GST-PCNA and His-SUMO-RAD18 recombinant proteins were purified from *E*. *coli*. For the NEDDylation assay, 1 μg GST-PCNA and 1 μg His-SUMO-RAD18 were incubated at 37°C for 1 h with E1, E2, NEDD8 in a total reaction volume of 20 μL following the product manual. Reaction was terminated by adding 20 μL 2× SDS loading buffer and detected by Western blotting.

### Immunofluorescence

HeLa cells were cultured on cover-glass in 6-well plates and transfected with the indicated plasmids for 24 h. Cells were then treated with 800 μmol/L H_2_O_2_ for 30 min, washed with 5 mL cold PBS, and lysed for 30 min in 0.2% Triton or NP-40 lysis buffer. Immunofluorescence staining was performed as described previously (Li et al., 2014). Cells were mounted with medium containing DAPI, and fluorescence images were obtained using a DuoScan microscope and LSM 710 NLO (Carl Zeiss, Oberkochen, Germany).

### Colony formation assay

Aliquots of 500 HeLa WT or *Rad18* ^−/−^ cells were treated with the indicated dose of H_2_O_2_ for 1 h and then cultured in 6-well plates for 12 days. Colonies were fixed and stained with crystal violet (Amresco, Solon, OH, USA), and the numbers of colonies were counted.

### Cell cytotoxicity assay

Aliquots of 10,000 HEK293T WT, *Nedp1* ^−/−^ or *Ubc12* ^−/−^ cells were cultured for 24 h in 96-well plates, then cells were treated with indicated dose of H_2_O_2_ for 1 h, or cisplatin and HU for 24 h. Next, cells were incubated with 90 μL medium and 10 μL CCK8 (Cell Counting Kit-8, Dojindo, Japan) for 4 h, then OD_450_ was measured using BioTek Cytation 5 (BioTek Instruments, USA).

### Bioinformatics analysis

Bioinformatics analyses were performed to analyze protein expressions of UBC12 and polη in patients of SKCM (skin cutaneous melanoma) using dataset of SKCM from GEPIA (gene expression profiling interactive analysis) (http://gepia.cancer-pku.cn/). And the correlation between the expression level of polη and SKCM prognosis was also analyzed.

## Electronic supplementary material

Below is the link to the electronic supplementary material.

Supplementary material 1 (PDF 890 kb)
