# Supplementary material for: NEDDylation antagonizes ubiquitination of proliferating cell nuclear antigen and regulates the recruitment of polymerase η in response to oxidative DNA damage
Source: Protein Cell. 2017 Aug 22;9(4):365–79. doi: 10.1007/s13238-017-0455-x (PMC5876183; doi:10.1007/s13238-017-0455-x)

Supplemental Information

NEDDylation antagonizes ubiquitination of Proliferating Cell Nuclear Antigen and regulates the recruitment of Polymerase η in response to oxidative DNA damage

Junhong Guan[1,2], Shuyu Yu[1,2], Xiaofeng Zheng[1,2*]

Fig. S1. PCNA is NEDDylated. (A) Immunoblotting analysis of PCNA NEDDylation in His-tagged NEDD8 conjugates enriched by Ni^{2+} pull-down (Ni-PD). (B) Analysis of the effect of NEDP1 on PCNA NEDDylation or its ubiquitination. In A-B, HEK293T cells were transfected with the indicated plasmids, and then PCNA NEDDylation or ubiquitination was examined by Ni-PD and western blotting with indicated antibodies. (C) Analysis of the interaction between RAD18 and UBC12. HEK293T cells were transfected with 3Flag-RAD18 and Myc-UBC12. At 48 h after transfection, cells were lysed in NP-40 lysis buffer and sonicated for 15 times. Co-IP was performed using antibody against anti-Flag or anti-Myc antibody. (D) *Ubc12*$^{-/-}$ HEK293T cells were generated using the CRISPR/CAS9 method. The expression of UBC12 in WT or *Ubc12*$^{-/-}$ HEK293T cells was analyzed by western blotting using anti-UBC12 antibody. (E) *Rad18*$^{-/-}$ HEK293T cells were generated using the TALEN method. The expression of RAD18 in WT or *Rad18*$^{-/-}$ HEK293T cells was analyzed by western blotting using anti-RAD18 antibody.

Fig. S2. NEDDylated PCNA accumulates in response to DNA damage. (A) A time course analysis of PCNA NEDDylation in His-tagged NEDD8 conjugates enriched by Ni-PD after UV treatment. (B) Analysis of the effect of UBC12-C111S on accumulation of NEDDylated PCNA under oxidative stress. In A and B, indicated plasmids were transfected into HEK293T cells and treated with 20 J/m^2 UV or 800 μM H_2O_2 for the indicated time. Then PCNA NEDDylation was detected by Ni-PD and western blotting with anti-PCNA antibody. (C) Analysis of the effect of MLN4924 on PCNA ubiquitination (upper panel) or its NEDDylation (lower panel) under oxidative stress. HEK293T cells were transfected with indicated plasmids and treated with DMSO or MLN4924, at 36 h after transfection, cells were treated with 800 μM H_2O_2 for the

indicated time. Then PCNA NEDDylation or ubiquitination was detected by Ni-PD and western blotting using anti-PCNA antibody. (D) A time course analysis of NEDDylated PCNA and ubiquitinated PCNA under UV-induced stress. HEK293T cells were transfected with indicated plasmids, after 36 h, cells were treated with 20 J/m^2 UV for the indicated time. Then His-tagged PCNA conjugates enriched by Ni-PD were detected by western blotting with anti-Myc, anti-HA or anti-PCNA antibodies.

Fig. S3. UBC12 knockout enhances PCNA ubiquitination. (A) Immunoblotting analysis of PCNA ubiquitination in HEK293T WT or *Ubc12*$^{-/-}$ cells. (B) Analysis of PCNA ubiquitination in HEK293T WT or *Ubc12*$^{-/-}$ cells under H_2O_2-induced oxidative stress. In A and B, indicated plasmids were transfected into HEK293T or *Ubc12*$^{-/-}$ cells and treated with or without 800 μM H_2O_2 for the indicated time. Then PCNA NEDDylation was detected by Ni-PD and western blotting with anti-HA (A) or anti-PCNA (B) antibodies.

Fig. S4. USP1 inhibits ubiquitination but not NEDDylation of PCNA. (A) Immunoblotting analysis of the effect of USP1 on PCNA NEDDylation or its ubiquitination. (B) Analysis of the effect of USP1 on PCNA ubiquitination under H_2O_2-induced oxidative stress. (C) Analysis of the effect of USP1 on PCNA NEDDylation under H_2O_2-induced oxidative stress. In A-C, indicated plasmids were transfected into HEK293 cells and treated with or without 800 μM H_2O_2 for the indicated time. Then PCNA NEDDylation or ubiquitination was detected by Ni-PD and western blotting with indicated antibodies.

Fig. S5. Polη is highly expressed in skin cancer patients and correlated to poor prognosis. Bioinformatics analyses were performed to study the expression of polη (A) and UBC12 (C) in SKCM (Skin Cutaneous Melanoma), and the correlation between polη expression and survival

in SKCM (B), using SKCM and normal dataset from GEPIA (gene expression profiling interactive analysis) (http://gepia.cancer-pku.cn/).

Fig. S6. Cropped and uncropped western blot images of Fig. 2E.

Fig. S7. Cropped and uncropped western blot images of Fig. 3A

Fig. S1

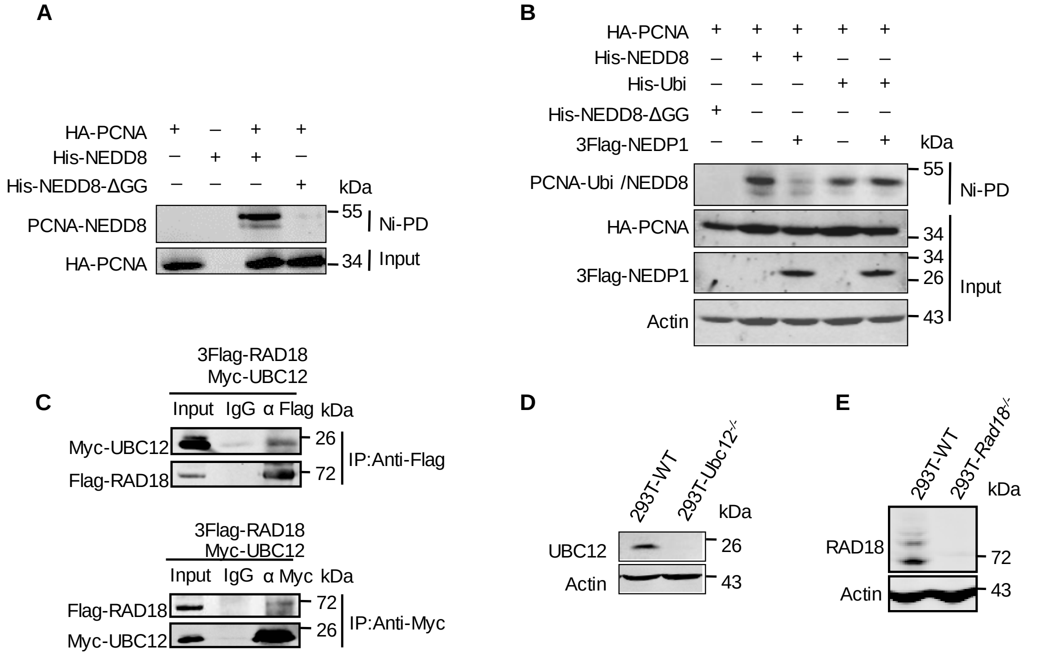
A
HA-PCNA
His-NEDD8
His-NEDD8-ΔGG
kDa
PCNA-NEDD8
HA-PCNA
Ni-PD
Input
B
HA-PCNA
His-NEDD8
His-Ubi
His-NEDD8-ΔGG
3Flag-NEDP1
PCNA-Ubi /NEDD8
HA-PCNA
3Flag-NEDP1
Actin
Ni-PD
Input
C
3Flag-RAD18
Myc-UBC12
Input IgG α Flag kDa
Myc-UBC12
Flag-RAD18
IP:Anti-Flag
Input IgG α Myc kDa
Flag-RAD18
Myc-UBC12
IP:Anti-Myc
D
293T-WT
293T-Ubc12-/-
UBC12
Actin
E
293T-WT
293T-Rad18-/-
RAD18
Actin

Fig. S2

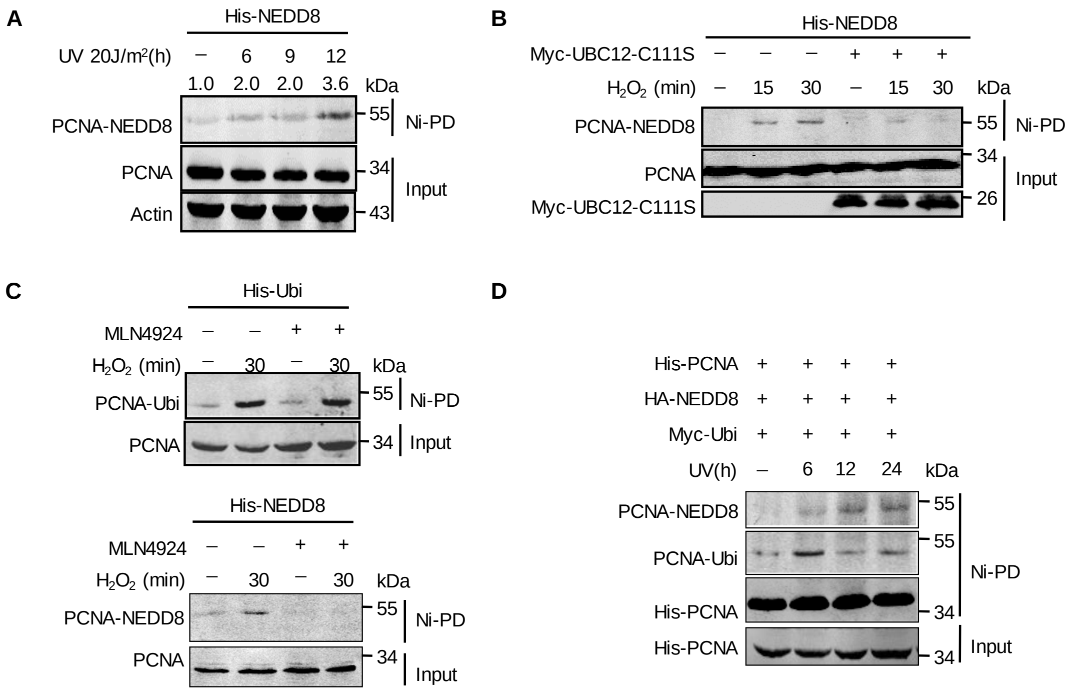
A
His-NEDD8
UV 20J/m2(h) – 6 9 12
1.0 2.0 2.0 3.6 kDa
PCNA-NEDD8
55
Ni-PD
PCNA
34
Input
Actin
43
B
His-NEDD8
Myc-UBC12-C111S – – – + + +
H2O2 (min) – 15 30 – 15 30 kDa
PCNA-NEDD8
55
Ni-PD
PCNA
34
Input
Myc-UBC12-C111S
26
C
His-Ubi
MLN4924 – – + +
H2O2 (min) – 30 – 30 kDa
PCNA-Ubi
55
Ni-PD
PCNA
34
Input
His-NEDD8
MLN4924 – – + +
H2O2 (min) – 30 – 30 kDa
PCNA-NEDD8
55
Ni-PD
PCNA
34
Input
D
His-PCNA + + + +
HA-NEDD8 + + + +
Myc-Ubi + + + +
UV(h) – 6 12 24 kDa
PCNA-NEDD8
55
PCNA-Ubi
55
Ni-PD
His-PCNA
34
His-PCNA
34
Input

Fig. S3

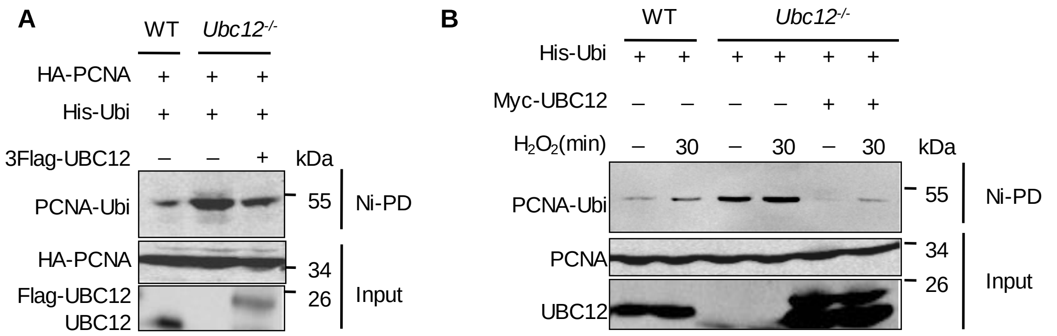
A
WT
Ubc12-/-
HA-PCNA
His-Ubi
3Flag-UBC12
kDa
PCNA-Ubi
55
Ni-PD
HA-PCNA
34
Flag-UBC12
UBC12
26
Input
B
WT
Ubc12-/-
His-Ubi
Myc-UBC12
H2O2(min)
30
kDa
PCNA-Ubi
55
Ni-PD
PCNA
34
26
UBC12
Input

Fig. S4

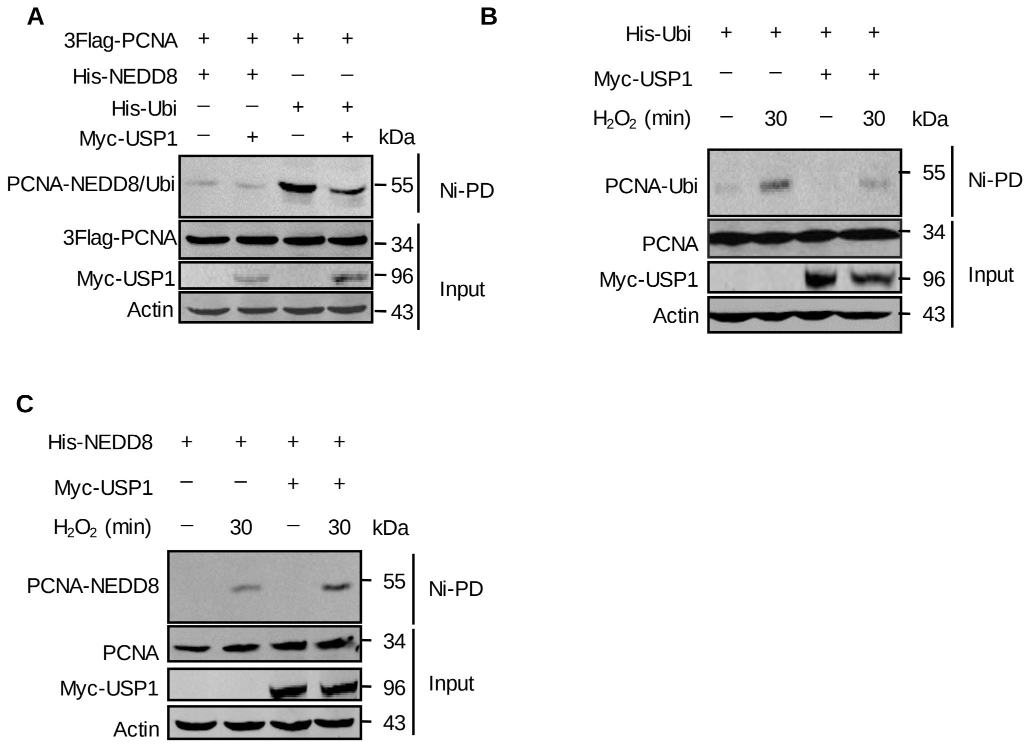
A
3Flag-PCNA + + + +
His-NEDD8 + + – –
His-Ubi – – + +
Myc-USP1 – + – + kDa
PCNA-NEDD8/Ubi
55
Ni-PD
3Flag-PCNA
34
Myc-USP1
96
Input
Actin
43
B
His-Ubi + + + +
Myc-USP1 – – + +
H_2O_2 (min) – 30 – 30 kDa
PCNA-Ubi
55
Ni-PD
PCNA
34
Myc-USP1
96
Input
Actin
43
C
His-NEDD8 + + + +
Myc-USP1 – – + +
H_2O_2 (min) – 30 – 30 kDa
PCNA-NEDD8
55
Ni-PD
PCNA
34
Myc-USP1
96
Input
Actin
43

Fig. S5

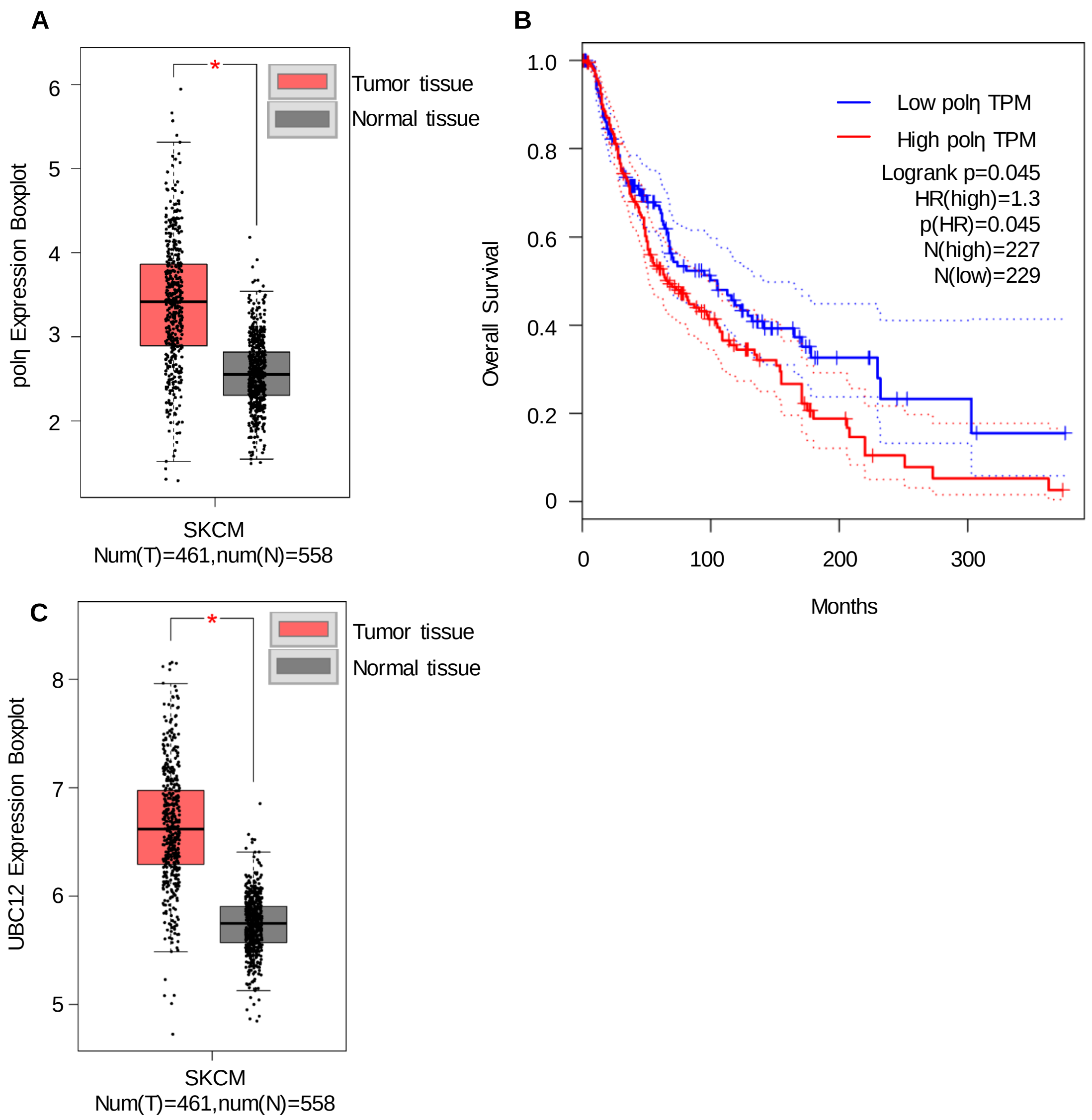
A
poln Expression Boxplot
6
5
4
3
2
*
Tumor tissue
Normal tissue
SKCM
Num(T)=461,num(N)=558
B
Overall Survival
1.0
0.8
0.6
0.4
0.2
0
Low poln TPM
High poln TPM
Logrank p=0.045
HR(high)=1.3
p(HR)=0.045
N(high)=227
N(low)=229
0
100
200
300
Months
C
UBC12 Expression Boxplot
8
7
6
5
*
Tumor tissue
Normal tissue
SKCM
Num(T)=461,num(N)=558

Fig. S6

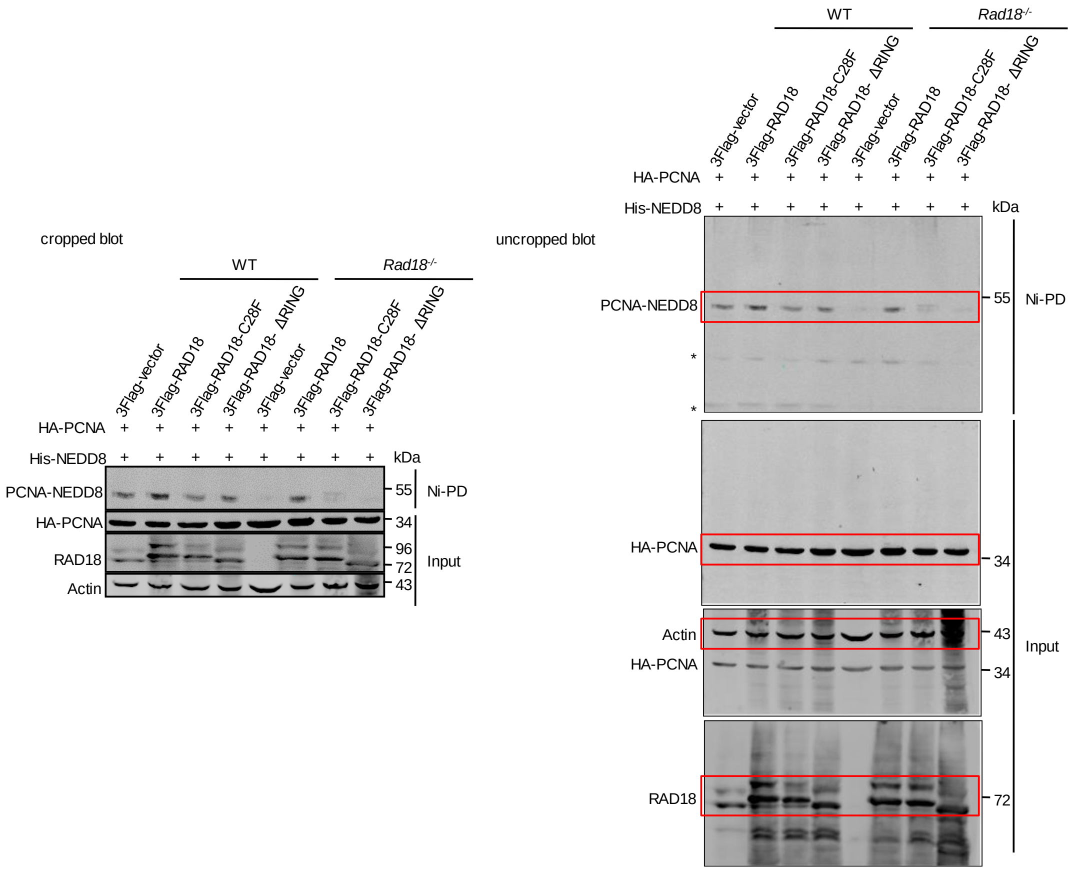
cropped blot
WT
Rad18-/-
3Flag-vector
3Flag-RAD18
3Flag-RAD18-C28F
3Flag-RAD18- ΔRING
HA-PCNA
His-NEDD8
kDa
PCNA-NEDD8
HA-PCNA
RAD18
Actin
55
34
96
72
43
Ni-PD
Input
uncropped blot
*
*

Fig. S7

cropped blot

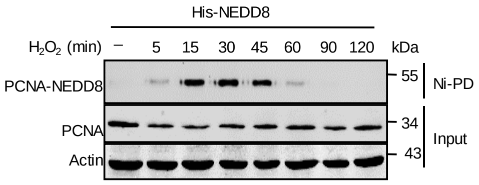

uncropped blot

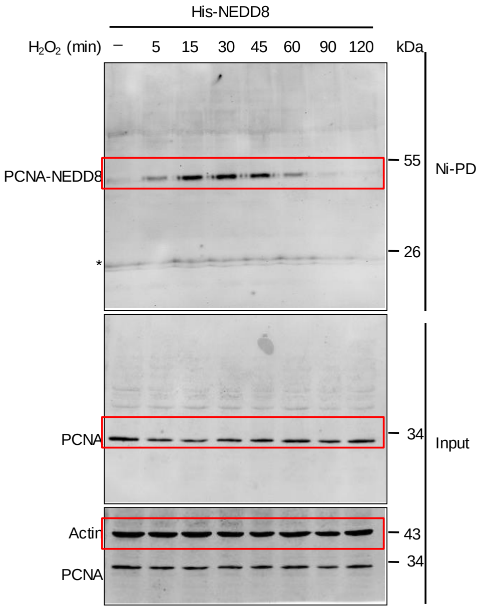

Supplement: Supplementary file 1 — Supplementary material 1 (PDF 890 kb) [file 13238_2017_455_MOESM1_ESM.pdf]
